# Urine TITIN N-fragment as a novel biomarker for critical illness myopathy: a pilot study

**DOI:** 10.1186/s13054-020-02842-5

**Published:** 2020-04-28

**Authors:** Hidehiko Nakano, Tsunehiro Matsubara, Kazuma Yamakawa, Kensuke Nakamura

**Affiliations:** 1 Department of Emergency and Critical Care Medicine, Hitachi General Hospital, 2-1-1 Jonancho Hitachi, Ibaraki, 317-0012 Japan; 2 Department of Traumatology and Acute Critical Medicine, Osaka University Graduate School of Medicine, 2-2 Yamadaoka, Suita-shi, Osaka, 565-0871 Japan; 3 Division of Trauma and Surgical Critical Care, Osaka General Medical Center, 3-1-56 Bandai-higashi, Sumiyoshi-ku, Osaka-shi, Osaka, 558-8558 Japan

## Main text

Intensive care unit (ICU)-acquired weakness is a muscular weakness without other cause after intensive care and consisting of critical illness polyneuropathy and critical illness myopathy (CIM) [1]. Muscle atrophy can occur quite early during critical illness because of increased muscle protein breakdown (MPB) and decreased muscle protein synthesis (MPS) [1]. Although muscle mass can be evaluated using ultrasound [2] and computed tomography (CT) [3], MPS and MPB are difficult to evaluate separately. Muscle biopsy can directly evaluate MPB in CIM directly [4], but it is invasive. Creatine kinase cannot evaluate MPB in CIM [1]. Therefore, no noninvasive technique has been established.

Titin, the largest protein expressed specifically in striated muscle, becomes various fragments that are released into blood and urine after muscle damage. Reportedly, the N-terminal fragments of titin (N-titin) excreted in the urine are increased in patients with various muscular diseases [5]. Therefore, N-titin is expected to be a noninvasive biomarker for monitoring MPB and we decided to evaluate the N-titin levels in the acute phase of critically ill patients.

Subjects were four adult patients admitted to our ICU in September 2019. We calculated the correlation coefficient between mean values of N-titin at days 1, 3, 5, and 7 and the femoral muscle mass (FMM) change between days 1 and 10 evaluated with CT. Urine N-titin levels were measured using an enzyme-linked immunosorbent assay kit (#27900 Human Titin N-Fragment Assay Kit, Immuno-Biological Laboratories Co. Ltd., Fujioka, Japan). FMM was calculated using the same method we previously reported [3]. Statistical analysis was done using software: R, ver. 3.6.1.

Subjects were four adults for whom activities of daily living before admission were sufficient for their independent living. Basic characteristics and outcomes of the respective patients are presented in Table 1.

**Table 1:** Basic characteristics and results for each patient

No.	Age	Sex	Diagnosis	SOFA	APACHEII	Hospital stay	ICU stay	Days on MV until day 10	Total steroids dose until day 10	RRT	Muscle relaxant use	Outcome	BI at day 28	CK (U/L)†	Cre (mg/dL)†	N-titin/Cre (pmol/mgCre/dL)*	FMM change (%)
**1**	86	F	Bacrerial pneumonia	6	15	20	9	5	PSL 150 mg	–	–	Alive	0	42.3	0.82	42.93	− 28.65
**2**	48	M	Interstitial pneumonia	6	9	91	15	10	PSL 360 mg mPSL 4000 mg	–	–	Alive	10	206.4	0.65	42.33	− 19.03
**3**	86	F	Congestive heart failure	7	20	11	5	2	PSL 180 mg	–	–	Alive	80	72.1	0.82	21.62	− 14.28
**4**	60	M	Malignant lymphoma	8	11	33	14	9	PSL 160 mg HYD 1200 mg	–	–	Alive	65	31.7	0.57	29.69	− 20.96

*SOFA* Sequential Organ Failure Assessment, *APACHE II* Acute Physiological and Chronic Health Evaluation II, *MV* mechanical ventilation, *RRT* renal replacement therapy, *BI* Barthel Index, *CK* creatine kinase, *N-titin* urine titin N-fragment, *Cre* serum creatinine, *FMM* femoral muscle mass, *PSL* prednisolone, *mPSL* methyl predonisolone, *HYD* hydrocortisone

*is for N-titin/Cre(pmol/mgCre/dL); ^†^is for CK and Cre

**Fig. 1:**
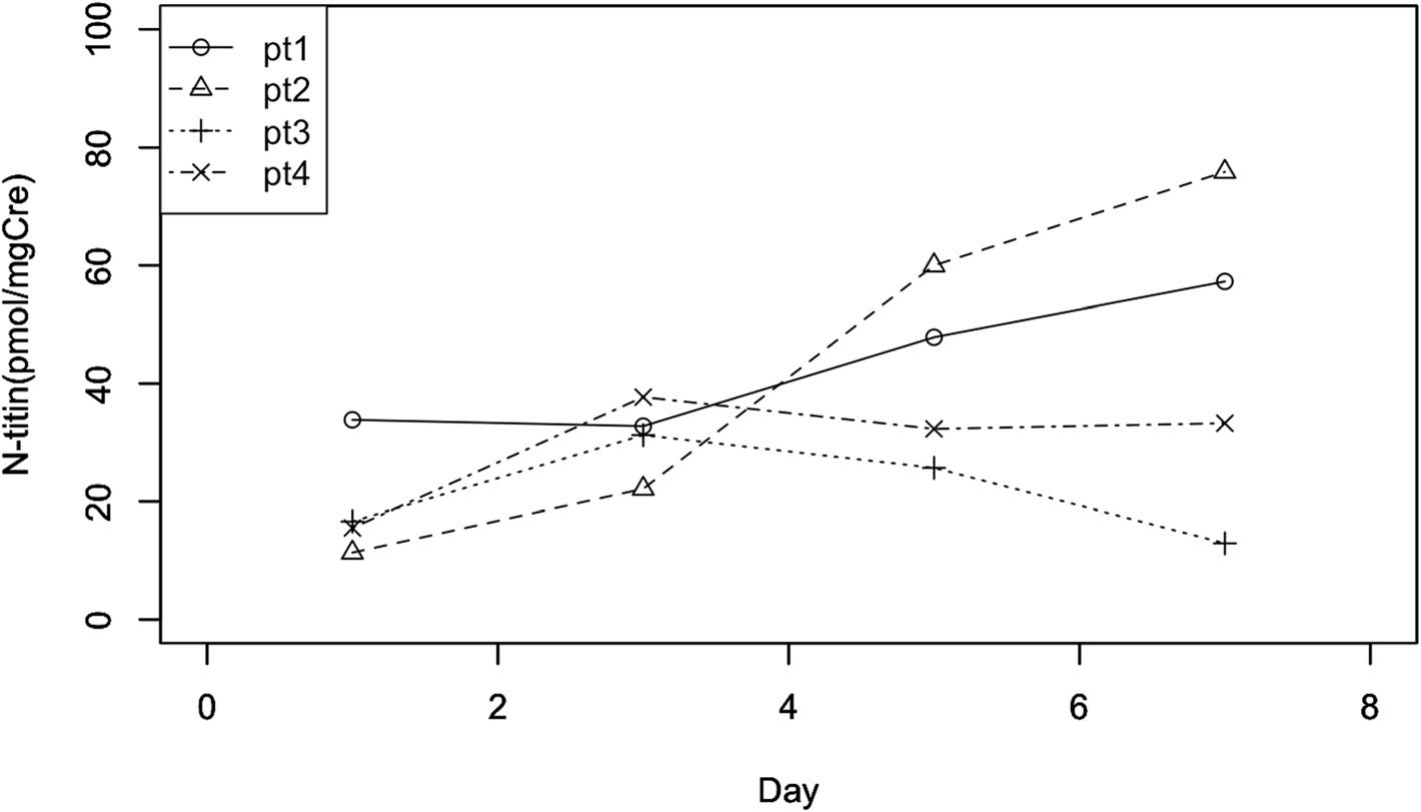
N-titin change of each patient. N-titin values were remarkably high when entering the ICU. They persisted for at least 7 days. N-titin showed continuous increase in patients 1 and 2 and peaked on day 3 in patients 3 and 4

Figure 1 shows N-titin changes of the respective patients. Mean N-titin levels of each patient were 42.93, 42.33, 21.62, and 29.69 (pmol/mgCre). Their respective FMM changes were − 28.65, − 19.03, − 14.28, and − 20.96 (%). The correlation coefficient between the mean value of N-titin level and the FMM change was negative: *r* = − 0.729. The mean values of creatine kinase for 10 days for the respective patients were 42.3, 206.4, 72.1, and 31.7 (U/L).

The N-titin values of critically ill patients were remarkably high when entering the ICU. They persisted for at least 7 days. The values were negatively correlated with the rate of FMM change. An earlier study [5] revealed that N-titin had a median of 1.2 (pmol/mgCre/dL) at rest in healthy individuals, 27.0 (pmol/mgCre/dL) at the highest after 10 km running, and a median of 735.5 (pmol/mgCre/dL) in patients with Duchenne muscular dystrophy. Although the N-titin levels of these patients were much lower than those of the muscular dystrophy patients, FMM decreased during a short duration. Not prominent but continuous active MPB persisting for a week and reducing MPS from anabolic resistance and malnutrition might have produced these results.

Another previous study [6] showed that N-titin levels in healthy individuals decrease during the first 2 weeks of bed rest. However, the present study showed high values in critically ill patients at ICU admission. These findings suggest that N-titin can reflect the pathological condition of MPB in CIM, which induced much more atrophy than muscle disuse.

No patient in this study was on dialysis. A molecular weight of N-titin is about 26 kDa, and N-titin is not removed by dialysis theoretically. Therefore, N-titin measurements are expected to be effective for patients on dialysis.

In conclusion, N-titin might be useful as a novel biomarker for MPB in CIM. For additional evaluation, a prospective observational study is underway at our hospital (UMIN000038353).

## Data Availability

The dataset is not available but can be requested from the corresponding author.

## Abbreviations

ICU: Intensive care unit
CIM: Critical illness myopathy
MPB: Muscle protein breakdown
MPS: Muscle protein synthesis
CT: Computed tomography
CK: Creatine kinase
N-titin: N-terminal fragments of titin
FMM: Femoral muscle mass
